# Locus-Specific Methylation of GSTP1, RNF219, and KIAA1539 Genes with Single Molecule Resolution in Cell-Free DNA from Healthy Donors and Prostate Tumor Patients: Application in Diagnostics

**DOI:** 10.3390/cancers13246234

**Published:** 2021-12-12

**Authors:** Olga Bryzgunova, Anna Bondar, Pavel Ruzankin, Petr Laktionov, Anton Tarasenko, Alexander Kurilshikov, Rostislav Epifanov, Marat Zaripov, Marsel Kabilov, Pavel Laktionov

**Affiliations:** 1 Institute of Chemical Biology and Fundamental Medicine, Siberian Branch of the Russian Academy of Sciences, 630090 Novosibirsk, Russia; a_bondar@niboch.nsc.ru (A.B.); a.m.kurilshchikov@umcg.nl (A.K.); somc@fmbansk.ru (M.Z.); kabilov@niboch.nsc.ru (M.K.); lakt@niboch.nsc.ru (P.L.); 2 Sobolev Institute of Mathematics, Siberian Branch of the Russian Academy of Sciences, 630090 Novosibirsk, Russia; ruzankin@math.nsc.ru (P.R.); tarasenko@math.nsc.ru (A.T.); 3 Department of Mathematics and Mechanics, Novosibirsk State University, 630090 Novosibirsk, Russia; 4 Epigenetics Laboratory, Department of Natural Sciences, Novosibirsk State University, 630090 Novosibirsk, Russia; laktionov@mcb.nsc.ru (P.L.); r.epifanov@g.nsu.ru (R.E.); 5 Institute of Molecular and Cellular Biology, Siberian Branch of the Russian Academy of Sciences, 630090 Novosibirsk, Russia

**Keywords:** cell-free DNA, methylation, locus-specific NGS sequencing, prostate cancer, benign prostatic hyperplasia

## Abstract

**Simple Summary:**

Prostate cancer (PCa) is the second most commonly diagnosed cancer in men, which is constantly accompanied by benign prostate hyperplasia (BPH). To reach a 100% 5-year survival rate in PCa, which is characteristic for PCa if it is diagnosed in early stages, efficient PCa diagnostics against the background of BPH are demanded. The article describes a liquid biopsy approach to differential PCa diagnostics based on the data on locus-specific methylation of the three genes (GSTP1, RNF219, and KIAA1539) obtained with NGS of cell-free DNA from blood plasma of PCa, BPH, and healthy individuals. We offered a diagnostic approach including the analysis of simultaneous methylation status of two CpGs in one cell-free DNA molecule, allowing the discrimination of all patients with absolute sensitivity and specificity.

**Abstract:**

The locus-specific methylation of three genes (GSTP1, RNF219, and KIAA1539 (also known as FAM214B)) in the blood plasma cell-free DNA (cfDNA) of 20 patients with prostate cancer (PCa), 18 healthy donors (HDs), and 17 patients with benign prostatic hyperplasia (BPH) was studied via the MiSeq platform. The methylation status of two CpGs within the same loci were used as the diagnostic feature for discriminating the patient groups. Many variables had good diagnostic characteristics, e.g., each of the variables GSTP1.C3.C9, GSTP1.C9, and GSTP1.C9.T17 demonstrated an 80% sensitivity at a 100% specificity for PCa patients vs. the others comparison. The analysis of RNF219 gene loci methylation allowed discriminating BPH patients with absolute sensitivity and specificity. The data on the methylation of the genes GSTP1 and RNF219 allowed discriminating PCa patients, as well as HDs, with absolute sensitivity and specificity. Thus, the data on the locus-specific methylation of cfDNA (with single-molecule resolution) combined with a diagnostic approach considering the simultaneous methylation of several CpGs in one locus enabled the discrimination of HD, BPH, and PCa patients.

## 1. Introduction

Prostate cancer (PCa) is the second most commonly diagnosed cancer in men. In 2018, PCa represented 13.5% of all cancers diagnosed in men [1]. PCa has a 100% 5-year relative survival rate if diagnosed in early stages of development, before distant metastases appear [2]. Therefore, screening for PCa in order to detect localized cancers is vital to the successful treatment of the associated pathologies and to increasing life expectancy and quality of life. The measurement of the level of prostate-specific antigen (PSA) in serum was included in the screening of PCa in the early 1990s all over the world, which led to an increase in incidence [3], but it simultaneously shifted this type of cancer to a predominantly localized form. However, according to a number of studies, the introduction of PSA screening causes the rate of overdiagnosis of PCa to reach almost 50%; moreover, when the threshold for PSA is in the range 2.5–4 ng/mL, the level of overdiagnosis can reach 80% [4]. As a consequence of false-positive results, patients have to undergo additional diagnostic procedures, including biopsy examinations [5], which can lead to pain, fever, bleeding, or urinary tract infections for one-third of patients [6]. However, according to a number of professional organizations, overdiagnosis can prolong the life of cancer patients and improve the quality of life, thus outweighing the disadvantages of side effects [7]. Since 2012, the U.S. Preventive Services Task Force (USPSTF) has no longer recommended PSA testing as a routine screening test for all men over the age of 50 [8]. In the same year, the “Progensa PCA3” test was approved by the Food and Drug Administration (FDA) for use in making re-biopsy decisions. PCA3 is a long non-coding RNA (lncRNA) that is overexpressed in prostate tissue, according to RT-qPCR data [9]. The “Progensa PCA3” test measures the ratio of PSA mRNA to PCA3 lncRNA in the analysis of cell sediments in urine collected after a rectal examination of the prostate [10]. This test has been shown to be more effective than measuring serum PSA levels [11,12]. However, when the data from 17 studies involving a total of more than 8600 participants were pooled in 2013 and the diagnostic scores for “Progensa PCA3” were determined, the PCA3 test failed to meet the high expectations. In those studies, the sensitivity ranged from 47% to 91% (pool mean: 69%), while the specificity ranged from 5% to 83% (pool mean: 58%), depending on the cutoff used [13]. Thus, the diagnostic characteristics of the method are far from being perfect, and therefore, the need to develop sensitive and specific tests for the diagnosis of PCa is still urgent.

Circulating blood cell-free DNA (cfDNA) is a convenient source of DNA originating from tumor cells and their environment and can be used to diagnose tumor development [14]. The most promising markers of oncotransformation are epigenetic changes in the composition of cfDNA, which primarily include the methylation of cytosines at the 5th position in CpG dinucleotides. Indeed, a change in the DNA methylation profile is one of the earliest markers of carcinogenesis [15,16]. In tumor cells, DNA methylation is impaired, including the hypermethylation of promoter regions of tumor suppression genes and the demethylation of regulatory regions of genes involved in oncogenesis [17,18]. Aberrantly methylated DNA was found in tumors in various tissues. In particular, an analysis of the methylation profile of the promoter region of the glutathione-S-transferase P1 (GSTP1) gene in the cfDNA pool can detect PCa with a specificity and a sensitivity of 89% and 52%, respectively [19]. The analysis of the methylation of the genes Septin 9 and SHOX2 in blood plasma cfDNA allows for detecting intestinal and lung cancer with a sensitivity and a specificity greater than 90% [20,21]. Both the Epi proColon^®^ and Epi proLung^®^ tests are approved for use as diagnostic tests in China and the European Union, and the former is also approved by the FDA in the United States (http://www.epigenomics.com, accessed on 11 December 2021).

The successes achieved by Epigenomics GA in the development of diagnostic systems confirm the promise of oncodiagnostics based on the analysis of aberrantly methylated blood cfDNA. However, the fact that there are only two diagnostic systems on the medical market demonstrates the difficulty of finding convenient tumor markers. This is primarily because tumor-specific aberrantly methylated DNA is detected in circulation much less frequently than in tumor tissue [22], due to its low concentration and the excess of “ballast” cfDNA in normal tissues. Actually, the bloodstream contains a common pool of DNA from all tissues of the body, and the similarity of methylation patterns in tumor cells to methylation patterns in distal normal tissues [23] can lead to an increase in false-positive test results. In addition, the detection of tumor-specific molecules in the blood is complicated by tissue-specific/age-specific/allele-specific DNA methylation [24,25].

The accurate measurement of the amount of aberrantly methylated DNA of a potential marker gene is also complicated by the phenomenon of heterogeneous methylation [25]. When creating diagnostic systems, DNA methylation status is determined within the framework of the PCR amplicons formed on a template converted by the bisulfite DNA [26]. The position of each CpG dinucleotide in each DNA template molecule can be either methylated or unmethylated (see illustration [25]). The biology of heterogeneous methylation is not fully understood [27]. Heterogeneous methylation profiles can appear as a result of tumor cell heterogeneity [28] due to their high variability [29], for example in the process of tumor development as a result of stochastic epigenetic aberrations at the “evolution” stage [30,31], as well as at the metastasis stage [31]. All these processes lead to the emergence of many tumor-specific molecules in the bloodstream, which means that the detection of individual aberrantly methylated molecules in the total pool of cfDNA becomes an extremely difficult task. For different donors, even with apparently identical types of tumors, the spectrum of epigenetic changes can be different [31]. The simplest situation pertains, when the mixture consists of completely methylated and completely unmethylated molecules; for example, when cells simultaneously contain these two variants of alleles [32]. The other case pertains, when the cells contain DNA methylated by several cytosines inside a single locus, and the positions of methylated cytosines differ between different DNA molecules (see illustration in [25]). In the former case, of a completely methylated or completely unmethylated DNA region, two independent systems (primers and sample) are sufficient for analyzing the DNA methylation status with the PCR method. In the latter case of heterogeneous methylation, i.e., in the presence of alleles with different methylation patterns, which we consider in this study, the detection of molecules with the desired methylation pattern is a much more complicated problem that cannot be solved using methyl-specific PCR.

Indeed, methods that only measure the methylation level of each CpG dinucleotide in the studied region for the entire pool of DNA molecules (pyrosequencing [33] or sequencing using primers independent of methylation [34]) do not allow obtaining data on the methylation profiles of individual molecules (see illustration in [25]). Heterogeneous methylation can be detected by the bisulfite sequencing of individual clones [35] or bisulfite DNA sequencing using NGS technology [31].

Despite the development of modern sequencing methods, the study of the DNA methylation of blood plasma originating from a tumor at the genome-wide level is a difficult task, due to the low concentration of cfDNA (10–100 ng/mL on average) [36,37], the low content of tumor-specific DNA (0.1–1% on average) [36,38], DNA degeneration during bisulfite conversion, and the possible errors in bisulfite conversion [39], as well as the variability in the methylation of tumor tissue alleles and tissue-specific methylation. This requires high-throughput sequencing (with multiple overlaps of the genome) and serious bioinformatics support. At the same time, the study of the methylation of individual loci via the method of locus-specific NGS-sequencing requires significantly fewer resources, in terms of both sequencing and bioinformatic and statistical analysis.

The aim of this work was to study the methylation of individual molecules in the blood’s cfDNA pool and to search for diagnostically significant methylation features that could be used for the differential diagnosis of prostate tumors.

## 2. Materials and Methods

### 2.1. Study Population and Blood Collection

Blood samples were taken from 17 patients with benign prostatic hyperplasia (BPH) and 20 patients with PCa, recruited at the Novosibirsk Regional Oncology Dispensary (Novosibirsk, Russia) (Table 1). A control group of 18 healthy donors (HD) (men with no evidence of prostate diseases, PSA level: <2.8 ng/mL) was recruited from ICBFM SB RAS clinics. Prostate biopsies and imaging techniques were applied to confirm the histopathological features and tumor stages of the PCa patients. Tumor staging was performed via TNM classification. All the PCa patients had tumors localized in the prostate without reported lymph node or distant metastasis (T2–3NxMx). None of the patients underwent treatment prior to or at the time of blood collection. The work was conducted in compliance with the principles of voluntariness and confidentiality in accordance with the “Fundamentals of Legislation on Health Care”. The study was approved by the ethics committees of ICBFM SB RAS and Novosibirsk Regional Oncology Center (N 15309-01 from 22.12.2008), and written informed consent was provided by all participants. Peripheral blood samples were collected into EDTA-containing Vacutainer tubes (BD, Cat. No. 368589) and fractionated into plasma and blood cells within 4 h of blood sampling. Plasma was collected from the blood samples after centrifugation at 400× *g* for 15 min and 800× *g* for 20 min and was frozen in aliquots at −20 °C until DNA isolation [40].

### 2.2. DNA Extraction and Characterization

Genomic DNA from human male leukocytes was used as an unmethylated DNA control (completely unmethylated DNA in the studied gene loci) to prepare the standards for TaqMan real-time PCR calibration curves.

Cell-free circulating DNA was extracted from 2 mL of blood plasma using a Blood Plasma DNA Isolation Kit (BioSilica Ltd., Novosibirsk, Russia) according to the manufacturer’s instructions, and this was eluted into 240 µL nuclease-free water. After the extraction, 225 µL of the DNA samples were mixed with 5 µL of glycogen (20 mg/mL, Fermentas, Vilnius, Lithuania) and a 1/10 volume of 50 mM triethylamine, and the mixture was then precipitated with 5 volumes of acetone [41]. After the precipitation, plasma cfDNA was reconstituted in 52 µL of water. The DNA aliquot derived in this step (2 µL) was diluted 6-fold and quantified by LINE 1 TaqMan PCR. The DNA samples were frozen and stored at −20 °C until use.

### 2.3. Bisulfite Conversion

Bisulfite treatment was performed using an EZ DNA Methylation-Gold™ Kit (ZymoResearch, Irvine, CA, USA) according to the manufacturer’s instructions. All the samples (50 μL) of circulating DNA (~10–30 ng) or similar amounts of genomic DNA were treated simultaneously. Bisulfite-treated cfDNA was eluted from DNA spin columns in 32 μL of an elution buffer. A DNA aliquot (2 µL) was used to quantify the DNA by LINE 1 TaqMan PCR, and 30 µL were stored at −20 °C until loci-specific PCR amplification. The sensitivity of LINE 1 TaqMan PCR was 50 pg of DNA, both before and after bisulfite treatment. Each sample used in the study contained at least 3.3 ng/mL DNA in the initial blood plasma, with a typical yield of cfDNA after bisulfite conversion varying between 70% and 80%.

### 2.4. TaqMan Real-Time PCR

TaqMan real-time PCR, targeting the LINE1 elements, was designed to amplify both the innate and the bisulfite-treated DNA in order to control the DNA loss after treatment, and its inputs into loci-specific PCR. Genomic DNAs from human male leukocytes before and after bisulfite conversion were used to derive calibration curves for different DNA samples. The total volume of the PCR was 30 μL using 5 μL DNA and a 15 μL 2× QuantiTect Kit. The reaction conditions are described in Table 2. Real-time PCR was performed on an iCycler iQ5 Real-Time PCR Detection System (Bio-Rad, Hercules, CA, USA).

### 2.5. Amplification of the Selected Loci

During the loci-specific PCR, the PCR products were barcoded with a pair of 8 bp unique indexes, with each encoding a patient. The barcodes were designed by Caporaso [42] to avoid errors in sequencing and data analysis.

The loci of interests were amplified using Hotstar Taq polymerase (Qiagen, Hilden, Germany), according to the manufacturer’s instructions, with minor changes. Briefly, 5 μL of bisulfite-treated cfDNA were amplified under the conditions described in Table 2. The unique barcodes are listed in Table 3. In order to exclude the appearance of chimeric DNA between the molecules of sick patients and HDs, the PCR products were coded using eight 5′ forward (Xn) and 12 reverse primers (Yn); as such, the groups of samples from PCa patients and those from HDs did not contain common barcodes. The sequences obtained with primer combinations that did not appear in the preparation of the libraries were considered chimeric and were excluded from the data analysis.

The reactions were performed using a Veriti Thermal Cycler (Applied Biosystems, Waltham, MA, USA). After the amplification, the PCR products were stored at −20 °C until use.

For each PCR, positive (5 ng genomic DNA from human male leukocytes after bisulfite conversion) and negative (no-template DNA) controls were used. The PCR products were quantified using a DNA 500 kit on the Shimadzu MCE-202 MultiNA (Shimadzu Corporation, Kyoto, Japan). After locus-specific amplification with methyl-independent primers, at least 50 ng DNA were obtained for each sample.

### 2.6. Preparation of the Sequencing Libraries

To prepare the DNA libraries, the PCR products of one gene and one patient group were pooled in equimolar amounts, as calculated using the data of the Shimadzu MCE-202 PCR product assay. Then, the PCR products were purified in 2% agarose gel using a GenJet Kit (Thermo Scientific, Waltham, MA, USA) and quantified using a Qubit fluorometer (Life Technologies, Carlsbad, CA, USA). At least 100 ng of the total PCR product mixture was used for the preparation of the library based on the Agilent 2100 Bioanalyzer data.

The DNA libraries were prepared using a NEBNext Ultra DNA Library Prep Kit (New England BioLabs, Ipswich, MA, USA), according to the manufacturer’s protocol. The library quality was evaluated with a High-Sensitivity DNA Agilent Chip run on the Agilent 2100 Bioanalyzer (Agilent Technologies, Santa Clara, CA, USA), which confirmed the size and concentration of PCR products. The libraries were analyzed via a 150 + 150 nt paired-end Illumina MiSeq sequencing run (Genomics Genetic Center, ICHBFM SB RAS). PCR products using completely methylated and completely unmethylated DNA were used as sequencing controls.

### 2.7. NGS Data and Statistical Analysis

The CpG methylation status of the DNA loci was analyzed with the BiQ Analyzer HT Software, using a minimal conversion rate of 0.95 and excluding reads with unrecognized sites.

To assess the representation of sequences with methylated and unmethylated cytosine in patients and healthy individuals, we introduced variables that corresponded to the proportions of such sequences. “C” stands for methylated cytosine, and “T” stands for unmethylated cytosine (uracil/thymidine after chemical conversion/amplification). The variables GSTP1.C1–GSTP1.C17 corresponded to the proportions (in the total number) of sequences displaying methylated cytosine in the investigated locus of the GSTP1 gene at positions 1 to 17, respectively. Similar variables were introduced for the loci of the RNF219 and KIAA1539 genes, i.e., RNF219.C1–RNF219.C17 and KIAA1539.C1–KIAA1539.C5.

To assess the sequences with methylated or unmethylated cytosine in two different positions of the same molecule, we introduced variables corresponding to the proportions of such sequences. For example, the variable RNF219.C4.T10 represented the proportion of sequences with methylated cytosine in the 4th position and unmethylated cytosine in the 10th position of the studied RNF219 locus. The methylation at one of the positions and pairwise “linked” methylation of the loci of genes GSTP1, RNF219, and KIAA1539 was described by 2 × (17 + 4 × (17 × 16/2)) + 5 + 4 × (5 × 4/2) = 1167 variables.

A statistical analysis of the distribution of variables in the following groups was performed:

1. Patients with PCa versus HD and BPH patients;
2. HD versus PCa and BPH patients;
3. BPH patients versus HD and PCa patients.

The distributions of the variables corresponding to one methylation position for the HD, BPH, and PCa groups were plotted in boxplots. The boxes depict the median and the 1st and 3rd quartiles.

For each of the introduced variables corresponding to methylation, the comparison groups were compared via the exact Mann–Whitney test. For the sake of convenience, we reported the adjusted *p*-values, which were the *p*-values multiplied by the number of comparisons (3 × 1167), according to Bonferroni’s approach. The differences corresponding to *p*-values < 0.05/(3 × 1167) (i.e., where the adjusted *p*-values were <0.05) were considered significant.

Besides *p*-values, we also calculated the following prediction accuracy measures for each comparison: sensitivity for a 100% specificity, specificity for a 100% sensitivity, accuracy for cross-validation, sensitivity for cross-validation, specificity for cross-validation, and the ROC AUC (Receiver Operating Characteristic curve with Area Under Curve) with a 95% DeLong’s confidence interval for cross-validation. The DeLong’s confidence intervals are not reported when the ROC AUC = 1, since in these cases, they are always (1, 1). We used leave-one-out cross-validation, and the values were computed via logistic regression with weights balancing the comparison groups. The cross-validation accuracy, sensitivity, and specificity were calculated with a threshold of 0.5. If complete separation (a 100% sensitivity for a 100% specificity of some cutoff value) was attained for a variable, then the accuracy, sensitivity, specificity, and ROC AUC were reported as 100%.

Additionally, the cutoff values were reported for the variables. The cutoff value was an estimate of the best threshold for separating the comparison groups. In the case of complete separation, the cutoff value was the geometric mean of the two closest values, with one being taken from one comparison group and the other from the other comparison group. If one of the two closest values was zero, then the arithmetic mean was reported instead. The reported ratio was the ratio of the two closest values, if neither of them was zero. If complete separation was not attained, then the cutoff was determined as the threshold that maximizes sensitivity + specificity and the ratio was not reported.

The mean values in the comparison groups were also reported. For the comparison of PCa vs. others, to explore the possibility of enhancing the prediction accuracy, we considered logistic regression models with multiple predictor variables. We used logistic regression with weights balancing the comparison groups. The variables were selected with forward selection based on the leave-one-out cross-validation prediction accuracy.

The analysis was performed with R v. 3.6.3 (R Core Team).

To check whether discriminative variables similar to those listed above might be found for low-coverage datasets, the whole-genome bisulfite sequencing (WGBS) data from the GEO database were used (GSE86832 and GSE104789) [43,44]. We used the WGBS data concerning the methylation patterns of primary prostate epithelial cells (GSE86832), normal prostate tissue, and prostate tumor biopsies (GSE104789). The reads were aligned on the human genome (GRCh38, Ensembl Release 103) using the Bismark bisulfite mapper, and non-converted reads were filtered out [45]. Reads mapped onto the loci of interest were extracted with Samtools [46]. Read post-processing was performed in Python. For the sake of simplicity, we used the same names for the new variables, but now the variables were binary and corresponded to the presence of a specific methylation pattern in each observed sequencing read. For the assessment of the associations of the variables with the comparison groups, the chi-square test was used wherein all the expected values were at least 5; otherwise, the exact Fisher test was used. The *p*-values were adjusted by multiplication by the number of comparisons 17 + 4 × (17 × 16/2) = 561. 

## 3. Results

The flowchart of the study is shown in graphical abstract.

The accuracy (variation coefficient) of the real-time PCR was 12%, the sensitivity was 5 pg of genomic DNA, and the efficacy was 97.4–99.5%.

Totals of 6.3, 9.0, and 9.2 million reads were obtained for GSTP1, RNF219, and KIAA1539, respectively, via 250 bp paired-end sequencing. The qualities of all libraries were high, and the percentage of uniquely mapped reads varied within the range 80–85%. The selected loci were sequenced with coverage ranging from 23,509 to 143,953.

After the primary data analysis, the number of molecules with similar methylation profiles for every gene in each patient was calculated.

**Figure 1:**
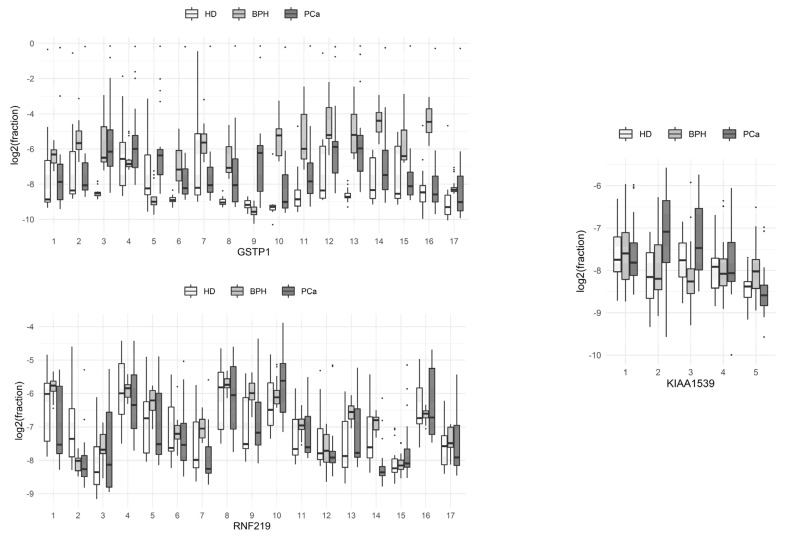
Comparison of the methylation levels of individual CpG dinucleotides at different positions of the studied loci of the genes of circulating blood DNA in HD, benign prostate hyperplasia (BPH), and prostate cancer (PCa) groups.

The proportions of sequences with methylated cytosine at each of the positions of the molecule in the HD, PCa, and BPH groups are presented in boxplots (Figure 1; boxes depict the median with the first and third quartiles).

From these data, it can be seen that C9 methylation at the studied locus of the GSTP1 gene was the most characteristic event associated with the development of prostate tumors, compared to methylation at other single positions of the gene locus. The methylation of the 3rd and 13th cytosines of the same locus significantly differed between HDs and BPH patients with prostate tumors. Patients with BPH and PCa differed most in terms of the methylation of the 2nd and 16th cytosines of the same locus. The total methylation levels of the individual positions of the RNF219 and KIAA1539 genes in the studied groups of patients did not differ significantly.

**Figure 2:**
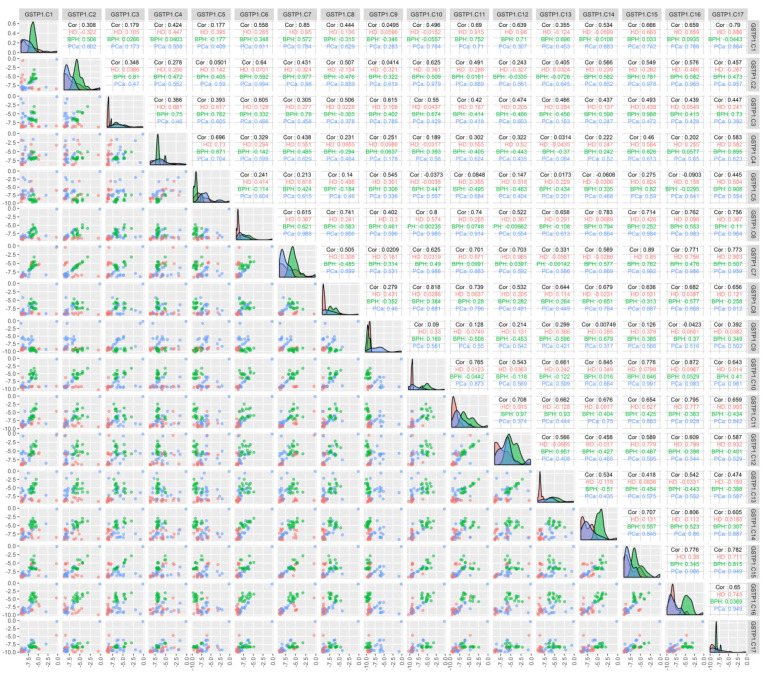
Correlation between the methylation of the individual cytosines of the studied locus of the GSTP1 gene and the composition of DNA in the circulating blood of the studied patients. The binary logarithms of the variables were used for all the plots. The diagonal cells present the density plots for the HD (red), BPH (green), and PCa (blue) groups for each of the variables. Each diagonal cell has its own *y*-axis scale, which is not labeled. “HD”, “BPH”, and “PCa” denote the respective Pearson correlations for each of the groups. “Cor” denotes the Pearson correlation for all the groups combined; the correlations were calculated for the binary logarithms of the variables. The cells below the diagonal present the scatterplots for each pair of variables.

An analysis of the correlation between the methylation of individual CpG dinucleotides for the GSTP1 gene (Figure 2) demonstrated that the methylation of individual cytosines correlates in HD and BPH patients (namely C7–C12 in HD patients and C3–C15 in BPH patients; r > 0.9), while for PCa patients, a high correlation (r > 0.9) was present for C2 methylation with C6, -7, -10, -15, and -17; C6 with C2, -7, -10, -11, -15, -16, and -17; and C10 with C15, -16, and -17.

The results of the comparisons between PCa and a group of HD and BPH patients are presented in Table 4. Only the variables with *p* × 3 × 1167 < 0.005 and a sensitivity (for a 100% specificity) of >70% are listed (the total data for *p* < 0.005 are listed in Table S1). In addition, the results for models with multiple predictor variables are listed as examples. There are dozens of other models with three predictors yielding a complete separation of the comparison groups (a 100% sensitivity with a 100% specificity). For instance, the following variables, together with GSTP1.C9 and RNF219.C2, displayed a complete separation of the comparison groups: GSTP.C2, GSTP.C16, GSTP.T2.T7, GSTP.T2.T9, GSTP.T2.T10, GSTP.T2.T11, GSTP.T2.T15, GSTP.T2.T16, GSTP.T7.T16, GSTP.T9.T16, GSTP.T10.T16, GSTP.T15.T16, GSTP.T1.C2, GSTP.T1.C16, GSTP.T2.C16, GSTP.T3.C16, GSTP.T4.C16, GSTP.T5.C16, GSTP.T6.C16, GSTP.T7.C16, GSTP.T8.C16, GSTP.T9.C16, GSTP.T10.C16, GSTP.T11.C16, GSTP.T12.C16, GSTP.T13.C16, GSTP.T14.C16, GSTP.T15.C16, GSTP.C2.T3, GSTP.C2.T4, GSTP.C2.T5, GSTP.C2.T6, GSTP.C2.T7, GSTP.C2.T8, GSTP.C2.T9, GSTP.C2.T10, GSTP.C2.T11, GSTP.C2.T12, GSTP.C2.T13, GSTP.C2.T14, GSTP.C2.T15, GSTP.C2.T16, GSTP.C2.T17, GSTP.C16.T17, RNF.T7.T9, RNF.C5.C6, RNF.C4.C13, KIAA.C2, KIAA.T2.T3, KIAA.T2.T4, KIAA.T1.C2, KIAA.C2.T3, KIAA.C2.T4, and KIAA.C2.T5. However, we are unsure whether the elevated prediction rate was a result of overfitting or if it reflected the true nature of the association. Therefore, we mentioned only a few models with multiple predictors. Moreover, many variables had good diagnostic characteristics, e.g., each of the variables GSTP1.C3.C9, GSTP1.C9, and GSTP1.C9.T17 demonstrated an 80% sensitivity at a 100% specificity. Note that the variables describing the methylation of the GSTP1 gene are of the greatest diagnostic significance.

**Figure 3:**
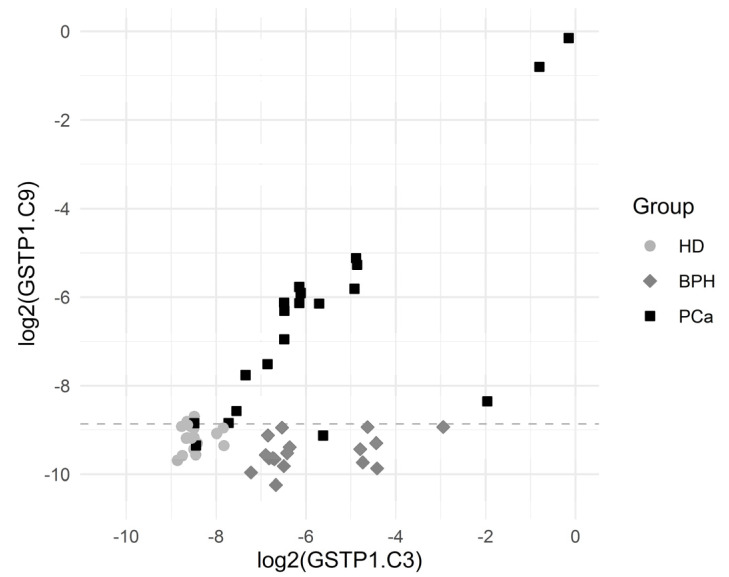
Scatterplots for GSTP1.C3 and GSTP1.C9. The horizontal dashed line depicts the cutoff value log2(0.00215) corresponding to a specificity of 94.3% and a sensitivity of 90.0% for the comparison of PCa vs. others.

The sensitivity (for a 100% specificity) of the other CpG positions within the GSTP1 gene locus was somewhat lower; however, the simultaneous use of data for the methylation of several CpG dinucleotides made it possible to construct diagnostic systems with a 100% sensitivity and a 100% specificity. Note that sequences methylated at diagnostically significant positions were well represented (3–8%), and the cutoff level varied in the range 0.1–0.2%. Figure 3 presents scatterplots for GSTP1.C3 and GSTP1.C9, which show that the BPH group was characterized by elevated GSTP1.C3 values (the values of GSTP1.C9 are normal) while the PCa group was characterized by the elevated values of both GSTP1.C3 and GSTP1.C9.

The cutoff value was an estimate for the threshold which may be good for separating the comparison groups.

Sensitivity was the proportion of PCa patients correctly identified as such, and specificity was the proportion of HD + PBH patients correctly identified as such.

Some of the results for the comparison of BPH patients with the group of HD and PCa patients are presented in Table 5. The variables with *p* × 3 × 1167 < 0.00000000010 and a 100% sensitivity for a 100% specificity are listed (the total data for *p* < 0.005 are listed in Table S2). The 80 variables (corresponding to two simultaneously methylated cytosines in each gene locus) allowed the complete discrimination of BPH patients, with *p* < 0.0000000001. Of those variables, 77 were derived from the RNF219 gene, and 3 are from GSTP1. Additionally, 46 of the variables discriminated the comparison groups, with a 94% specificity for a 100% sensitivity and with *p* < 0.00001.

**Figure 4:**
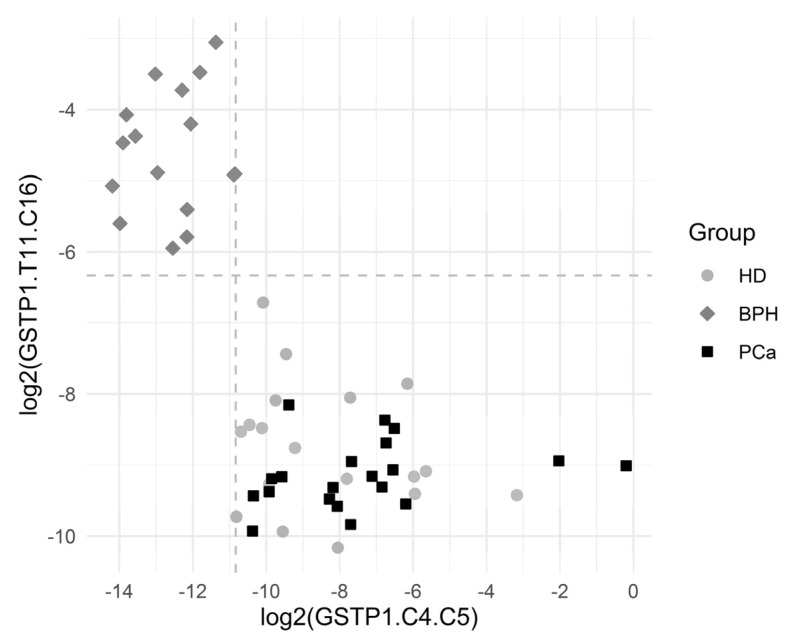
Graphical representation of the BPH group separation. The horizontal dashed line depicts the cutoff value log2(0.0214). The vertical dashed line depicts the cutoff value log2(0.000548). Both the cutoff values correspond to the complete separation of BPH vs. others for the respective variables. One observation from the BPH group with log2(GSTP1.C4.C5) = 0 and log2(GSTP1.T11.C16) = −3.1 is not depicted, since log2(GSTP1.C4.C5) is infinite for that point.

Figure 4 depicts the separation of the BPH group via GSTP1.C4.C5 and GSTP1.T11.C16. The results showed a statistically significant association between many of the introduced variables and the BHP outcome.

The cutoff value was an estimate for the threshold which may be good for separating the comparison groups. The corresponding ratio was reported in the case of complete separation, which was the ratio of the minimum in the comparison group with higher values to the maximum in the comparison group with lower values.

Sensitivity was the proportion of BPH patients correctly identified as such, and specificity was the proportion of HD + PCa patients correctly identified as such.

The results of the comparison of HDs with BPH and PCa patients are presented in Table 6. The variables with *p* × 3 × 1167 < 0.000000035 are listed (the total data for *p* < 0.005 are listed in Table S3). Only the variable GSTP1.T3.T13 allowed the complete separation of HDs from patients with prostate tumors. There were seven variables with sensitivity of 77–89% for a 100% specificity.

The frequency of occurrence of molecules with diagnostically significant sites in the blood plasma of HD was 0.2–1%, and that was 1–11% for the other groups. The diagnostic level was 0.5–1%; hence, the detection of such differences did not require special approaches or highly sensitive analytical systems.

**Figure 5:**
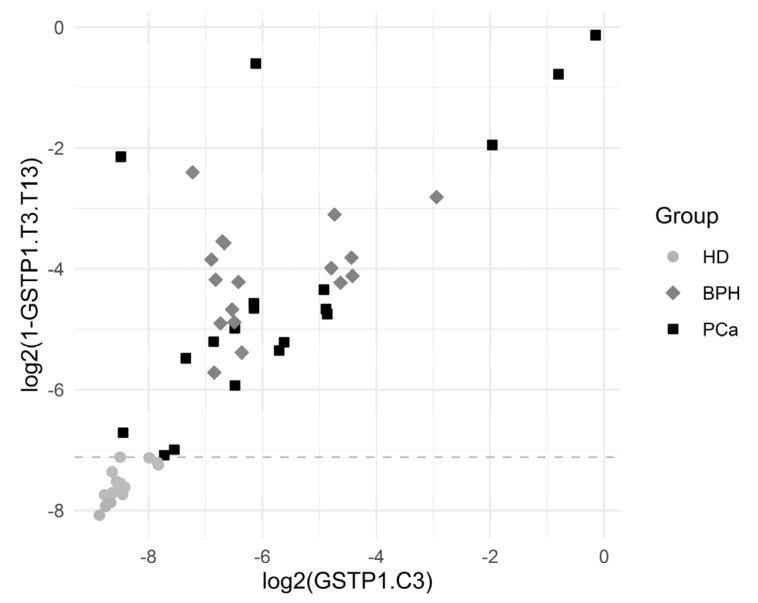
Graphical representation of HD separation by GSTP1.T3.T13. The horizontal dashed line was plotted for the cutoff value log2(1 − 0.99281), which corresponded to the complete separation of HDs from the others.

Figure 5 depicts the separation of the HD group by the variables GSTP1.T3.T13 and GSTP1.C3. The results showed a statistically significant association between many of the introduced variables and the HD outcome.

The cutoff value was an estimate for the threshold which may be good for separating the comparison groups. The corresponding ratio was reported in the case of complete separation, which was the ratio of the minimum in the comparison group with higher values to the maximum in the comparison group with lower values.

Sensitivity was the proportion of HD correctly identified as such, and specificity was the proportion of BPH + PCa patients correctly identified as such.

To check whether diagnostically relevant methylation patterns might be found in independent studies, we decided to test the same approach on published whole-genome bisulfite sequencing (WGBS) datasets in PCa samples. The DNA methylation microarray, as a technology used to analyze genome-wide methylation patterns in pathology and bisulfite sequencing data, is rather scarce. To perform our analysis, we used the WGBS of DNA samples of benign normal prostate and prostate tumor biopsies (GSE104789) and the WGBS of primary prostate epithelial cells’ DNA (PrEC) (GSE86832) [43,44]. In total, we analyzed WGBS datasets for three benign normal prostate samples, 11 prostate tumor samples (both from GSE104789), and four primary prostate epithelial cell samples (GSE86832). In fact, we were able to extract sequences only for the investigated locus of the GSTP1 gene from the datasets used, while the loci in the RNF219 and KIAA1539 genes were covered by single reads and had to be omitted from analysis. The coverage of the GSTP1 gene locus was also non-uniform due to the rather short sequencing reads—paired-end 70 bp for the GSE104789 dataset and paired-end 100 bp for the GSE86832 dataset. Each sample from the GSE104789 dataset contained 12 to 99 sequenced fragments (the median value was 40) in the GSTP1 gene locus, with a median length of around 100 bp. The samples from the GSE86832 dataset contained 6 to 10 sequenced fragments in the same locus (median value: 8.5), with a median length of around 140 bp. All the reads overlapping the investigated locus of the GSTP1 gene were pooled into the two groups, i.e., the normal group (prostate biopsy samples and primary prostate epithelial cell samples) and the cancer group (prostate tumor biopsy). To assess the associations between the group and the methylation pattern, we used variables similar to those introduced before.

Here, we used the same names for the new variables, but now the variables were binary and corresponded to the presence of a specific methylation pattern in each observed sequencing read. Due to a large proportion of missing data, the numbers of available reads differed between the variables.

The comparison of the normal group and the cancer group via the WGBS data allowed us to detect significant differences in 121 variables (*p* < 0.05; Table S4). Among these variables, 24 were detected in comparison of HDs with joint groups, including patients with prostate tumors, as mentioned above (Table S5). The analysis of the WGBS data allowed us to detect the GSTP1.T3.T13 variable, which provided a complete separation of HDs from patients with prostate tumors (Table 6). The top five variables (GSTP1.T3.T13, GSTP1.T8.T13, GSTP1.T9.T13, GSTP1.T6.T13, and GSTP1.C13) of the seven with a sensitivity of 77–89% for a 100% specificity, listed in Table 6, were represented significantly differently between the normal and cancer groups in terms of the WGBS data. Two variables (GSTP1.T5.T9 and GSTP1.C9) that might be used for discriminating between PCa patients and the group of HD and BPH patients were found (Table S6) in these datasets. It should be noted that short reads and low coverage levels (the only few of 17 CpGs were covered) precluded the identification of even major methylation profiles. Nevertheless, these characteristic patterns were found in independent datasets and, in general, were proportional to their representation in locus-specific methylation data.

## 4. Discussion

PCa is well-known for its association with the epigenetic alterations underlying its development [47]. This makes studying DNA methylation (including that in liquid biopsies) one of the most promising approaches in tumor biomarker research [48]. Nevertheless, until now, no existing genetic diagnostic test for PCa has been able to outperform routine PSA screening [49], despite the development of the PCA3 test and several others [50,51].

The methylation index, i.e., the averaged ratio of methylated to unmethylated CpG, displayed low specificity when analyzed in tissue samples, plasma or urine. However, such early works helped to identify the list of potentially useful genes, e.g., GSTP1, and SFRP2 [52,53]. In the microarray study by the Petronis laboratory, several new markers of aberrant methylation were identified [54]. Undoubtedly, NGS provides the most accurate and comprehensive information regarding the methylation status of individual DNA molecules.

In this study, we have analyzed methylation on the resolution level of individual CpG dinucleotides, employing locus-specific NGS sequencing in order to reveal aberrantly methylated DNA circulating in the blood that is specific to prostate tumors. The three genes, i.e., GSTP1, RNF219, and KIAA1539, which are shown to change the methylation status of prostate tumor patients [34,54,55], were studied. Previously, it was believed that the methylation of the promoter region of the GSTP1 gene in the genomic DNA isolated from prostate tumor tissue was close to 100%; however, using bisulfite sequencing, it was shown that this gene is methylated in 86% of prostate tumor tissues [56]. An analysis of the aberrant methylation of the RNF219 gene in the blood plasma of patients allowed distinguishing PCa patients from BPH patients with a sensitivity of 61% and a specificity of 71% [54]; the good potential diagnostic properties of the KIAA1539 gene were also demonstrated in [54].

On average, 1 mL of plasma from a patient with cancer contains approximately 1500 diploid genome equivalents (GEs) (~10 ng DNA), with considerably higher amounts often observed in patients with metastatic cancer. A typical 10 mL blood draw yields, on average, 4 mL plasma containing 6000 GEs (12 × 103 molecules per region or gene), which implies a theoretical sensitivity limit of ~0.01% (that is, the ability to detect 1 fragment in 12,000 copies) [36].

Considering the proportion of tumor-specific DNA in circulation [57], even 104 reads per locus per sample provide at least 100× coverage of cfDNA and reliable detection of a single tumor-specific molecule.

The analysis of the methylation data of individual cytosines within the studied loci revealed two positions, C9 and C3, in the GSTP1 gene that differed between the studied groups. However, the sensitivity and specificity in detecting patients with PCa did not exceed 90% and 94%, respectively. At the same time, no such positions were found in the studied loci of other genes. In addition to the methylation of individual cytosines within the studied loci, we considered the pairwise methylation of molecules or, more precisely, introduced variables that reflected simultaneous methylation, the methylation of one and no methylation of the second, or the absence of methylation of the two cytosines in one DNA molecule. This approach helped us to identify potential markers that discriminated the comparison groups with a 100% sensitivity and specificity. It was possible to discriminate HDs efficiently using a single methylation profile of the GSTP1 molecule, while the absolute detection of patients with PCa required the simultaneous detection of at least three profiles from the loci of the GSTP1 or RNF219 genes. It should be noted that, for PCa diagnostics, the DNA sites were mainly methylated, whereas for the discernment of a healthy person, they were unmethylated. However, we were not sure about whether the high prediction rate was a result of overfitting or it reflected the true nature of the association. Therefore, we mentioned only a few models with multiple predictors. Nevertheless, the results showed a statistically significant association between many of the introduced variables corresponding to the methylation and the PCa outcome.

When analyzing the data on the frequency of representation of molecules with a marker methylation profile in circulation and the cutoff level (Table 4), the representation of molecules with such sequences is generally close to the percentage of tumor-specific DNA in circulation [36,57]. The diagnostic level permits the reliable detection of differences between tumor patients and healthy people using standard approaches. Indeed, NGS with a coverage, at a single locus, of 10,000 or more should already allow the reliable detection of the difference between sickness and health, in addition to PCR with a single nucleotide extension of the primer, with the subsequent detection of PCR products using mass spectrometry (for example, Agena Bioscience can detect 1% variant allele frequency, starting with 10–15 ng DNA (https://agenabio.com/products/panels/cancer-solutions/, accessed on 3 July 2021).

For the detection of BPH, analyzing the methylation of the RNF219 gene seemed to be the most feasible approach. Indeed, the presence of the 79 variables (corresponding to two simultaneous methylated cytosines in one molecule) exclusively discerning BPH patients, with adjusted *p* < 0.0000000001, suggested that an increase in sample size will not change the situation and at least some of the variables will retain their diagnostic efficacy in single-molecule analysis or in an analysis of a set of diagnostic molecules close to those previously described for miRNA [58]. It should be noted that, as of now, the absolute discrimination of BPH patients remains one of the hardest problems. Practically all patients with PCa have areas of prostate tissue with hyperplasia. The phenomenon of loci methylation that is typical for BPH RNF219 is an unexpected gift for PCa differential diagnostics, and it also demonstrates the principal importance of this gene in the transition from non-malignant to malignant tumors. Considering its more or less uniform expression in most tissues (with higher levels in the testis; https://www.proteinatlas.org/ENSG00000152193-RNF219/tissue, accessed on 3 July 2021), figuring out the role of this protein in malignant tumor transition is very exciting.

In the group of BPH patients, we observed practically no methylation of the positions in the RNF219 gene, i.e., the level of their methylation did not exceed 0.02%. The diagnostic threshold is near 0.06%. Thus, a more sensitive analytical system is needed in order to identify patients with BPH than the system used for identifying patients with PCa. Nevertheless, a large array of data that allow for discriminating between patients and healthy people with a 100% sensitivity and specificity provides great opportunities for choosing the best analytical systems.

Several platforms for detecting aberrantly methylated DNA are available, such as Illumina HumanMethylation 450 BeadChip array, DREAMing, and Illumina HumanMethylation27 Bead-Chip [59,60,61,62,63,64,65], but only NGS reports accurate information regarding the methylation status of each cytosine of the DNA loci. In order to reveal the simultaneous methylation of two cytosines in one DNA loci in clinical diagnostics, next-generation MPS combined with target enrichment protocols [66] can be employed, as well as mass-spectrometry-based platforms, such as MassARRAY from Agena Bioscience Inc. Actually, the last platform allows the fast and cost-efficient visualization of cytosines in given positions and can be modified to perform simultaneous cytosine localizations. Considering the tendencies of decreasing sample prices and the expanding applicability of both protocols in clinics, realizing the detection of aberrantly methylated profiles of specific loci seems likely. For example, Illumina announced a multiplexed DNA methylation analysis protocol, which allows for the simultaneous quantitative measurement of cytosine methylation, at the individual-nucleotide resolution, in 48 PCR amplicons and 48 samples utilizing the microfluidic system established by Fluidigm [67]. Following the bisulfite conversion of the target DNA, a PCR is performed using a 48.48 access array, which allows the parallel amplification of 48 samples by 48 primer pairs. The products of each reaction are labeled with individual sample-specific tags and then pooled in a single library and sequenced.

There are few additional advantages to assessing methylated DNA as a cancer biomarker, since the phenomenon of DNA methylation in tumors is widespread [68,69]. In contrast to miRNA, aberrantly methylated DNA is a much more “precise” marker, since it directly regulates only one gene, but is not involved in gene net regulation [70]. Besides this, working with DNA is favorable for RNA assays, not only due to the importance of DNA methylation in genome regulation, but also because DNA is more stable, thereby simplifying sample collection, processing, transport, and storage. Another advantage is that when performing assays in heterogeneous cell populations, the amount of DNA extracted is proportional to the cell count. On the contrary, for RNA, a small subpopulation of cells with high transcription rates can mask the profiles of the others [71]. Next, even if at first glance the data for locus-specific methylation obtained by NGS may look over-informative, the correlation of the DNA data with that on tumor development, therapy efficacy, and tumor relapse can be used to increase the efficacy of tumor treatment.

It should be noted that the number of profiles obtained in this study exceeded the level of estimated data obtained from the cfDNA concentration, and this may be related to the sequencing technology used. In other words, the obtained data need verification via reference methods. Nevertheless, a large set of data for verification suggests that at least some of the detected markers will possess the declared characteristics and thus improve the accuracy of diagnosis in PCa.

The promise of using methylated cfDNA as a source of tumor markers is also raised by its increased stability compared to unmethylated DNA, which allows methylated DNA to circulate in the blood for a much longer time [72]. This, in turn, should lead to an increase in the amount of the desired tumor marker in the blood with the growth of the tumor and, accordingly, to an increase in the accuracy of detection of the disease. In view of the increasing spreading of NGS-sequencing in medical practice, the development of the corresponding devices and the emergence of sequencers aimed at clinical use, the availability of this type of tests in clinics in the nearest future look real. The tests could find applications to differential diagnostics of prostate tumors as well as to monitoring of the PCa patients after therapy. However, in order to achieve that lofty goal, a number of experiments, including those expanding the prostate tumor patients’ number as well as involving patients with inflammatory prostate diseases, must be conducted. Since the characteristic features of marker sequence methylation (simultaneous methylation of cytosines of the studied loci) that we discovered may reflect both the features of the regulation of gene expression and the features of DNA methylation in PCa patients (Figure 2), the effect of methylation profile on gene expression is desired to be studied. For example, methylation at the 7th and 12th, as well as at the 3rd and 15th, cytosines may not be essential for the inhibition of GSTP1 gene expression. On the other hand, methylation at the positions listed for patients with PCa can lead to the inhibition of gene expression, which has been shown for malignant PCa tumors [52,73], and can also reflect both the peculiarities of DNA packaging and the work done by tumor cell methylases.

The latest results on this topic were reported at the ESMO Congress by Oxnard et al. and were shown to be promising when used for detecting methylation signatures for the early detection of multiple cancer types (https://grail.com/wp-content/uploads/ESMO_2019_Oxnard_CCGA2_Training_Final.pdf, accessed on 3 July 2021). The data from 4500 patients proved the utility of targeting the most informative regions of the genome and were used in machine learning algorithms to detect the presence of cancer and identify the tumor’s tissue of origin. The results of our study, which were obtained from a rather small sample and focused on single regions, coincide with the described findings from large-scale and genome-wide studies. This suggests that DNA methylation analysis is a powerful diagnostic tool and increases the need for further research in the field.

The analysis of the WGBS data allowed us to extract methylation patterns associated with PCa in low-coverage data. The genome coverage in the dataset used was quite low and allowed us to extract sufficient data for the GSTP1 gene locus only. This was expected, as the well-known shortcomings of WGBS data include their low coverage, especially when compared to targeted bisulfite sequencing, and the usage of short sequencing reads, together leading to missing values in the regions analyzed and making it nearly impossible to simultaneously analyze CpGs from single DNA molecules located more than 150 bp apart. Taking into account the drastically different coverage for each of the loci analyzed in our targeted bisulfite sequencing experiments and in the open WGBS datasets, specific results for the methylation patterns observed should not be expected. However, around 20% (26 out of 121) of the variables that were significantly different between the normal and cancer groups, according to the WGBS data, were determined to be diagnostically significant in our targeted bisulfite sequencing (BS-seq) experiments. It is important to note that 24 of the variables produced by WGBS that overlapped with those derived from targeted BS-seq comparisons of HDs with a joint group containing patients with prostate tumors showed the same tendency to be over-represented in healthy and cancerous samples (Table S5). The difference in the representation of the variables in each group was even greater in the WGBS data as compared to the targeted BS-seq data, which might be explained by the type of samples analyzed (Table S5). Indeed, biopsy samples and primary cell culture were used for WGBS, which reflected discrete methylation patterns in normal and cancer cells, whereas the pathological cfDNA samples used for the targeted BS-seq experiments are inevitably loaded with normal cells’ DNA. Although the biopsy samples might contain normal cells as well, in general, the DNA biopsy samples used for analysis were enriched in tumor cell DNA as compared to in cfDNA. Two variables that distinguished PCa, as derived from the targeted BS-seq experiments, were found to be significantly differently represented between normal and cancerous samples in the WGBS experiments, namely, GSTP1.T5.T9 (88.6/99.1%—averages in the PCa/healthy and BPH groups, targeted BS-seq; 15.0/54.5%—positive in the cancer/normal groups according to WGBS) and GSTP1.C9 (8.35/0.160%—averages in the PCa/healthy and BPH groups according to the targeted BS-seq; 79.9/51.8%—positive in the cancer/normal groups according to WGBS) (Table S6). Although due to the intrinsic limitations of methodology and different sources of the analyzed biomaterial, WGBS data analysis is far from being optimal to independently validate the targeted BS sequencing results. Nonetheless, a significant portion of discriminative variables detected in WGBS study was found to be diagnostically significant in the targeted approach. Therefore, it is tempting to speculate that the use of the described concept for the analysis of whole-genome datasets might help to identify candidates of differentially methylated genomic regions whose diagnostics potential might be further investigated with the targeted sequencing approaches on donor cohorts.

## 5. Conclusions

Thus, using the locus-specific MPS method, we derived data on the methylation of the RNF219, KIAA1539, and GSTP1 gene regions in the blood plasma cfDNA of patients with PCa and BPH and in HDs. It was shown that, using these data, it is potentially possible to classify the patients of all the studied groups with a 100% sensitivity and specificity.

The obtained data on the correlation of the methylation of CpG dinucleotides with the composition of the studied loci not only have diagnostic value and are applicable for personalized medicine, but also suggest that such methylation is fundamental to the regulation of the expression of the studied genes (since the expression reliably correlates with the development of the tumor) and to tumor development.

At the same time, we considered the differentiation of PCa from BPH as a strong feature of the proposed approach, since malignant prostate tumors are accompanied with hyperplasia of prostate tissue and an accurate discrimination of BPH from PCa is still demanded in practical medicine.

## Figures and Tables

**Table 1:** Overview of the study population.

Characteristic	Groups		
	Prostate Cancer Patients	Benign Prostatic Hyperplasia Patients	Healthy Donors
	(*n* = 20)	(*n* = 17)	(*n* = 18)
Age			
Mean ± SD	67.6 ± 7.3	66.6 ± 7.5	61.7 ± 6.6
Range	55–77	54–79	53–74
Total PSA (ng/mL)			
Mean ± SD	17.5 ± 12.3	10.7 ± 8.5	1.2 ± 0.7
Range	4.8–48.7	0.6–41.5	0.2–2.3
Tumor stage			
T2bNxMx	7	N/A	N/A
T2cNxMx	6		
T3aNxMx	4		
T3bNxMx	3		
Gleason scale			
Unknown	1	N/A	N/A
4–5	1		
5	2		
5–6	3		
6	7		
7	4		
8	2		

**Table 2:** PCR conditions and primers’ sequences.

Target’s Name	Primer’s Sequence (without Barcodes)	Primers/Probe Concentration (nM)	Length of PCR Product (bp)	Length of PCR Product without Barcodes (bp)		CG Number	1× Buffer Composition	PCR Conditions	
LINE1-For LINE1-Rev LINE1-Probe	5′-AATGGAAGATGAAATGAATGAAATGA-3′	600/ 300	-		155	-	BioMaster qPCR Mix from Biolabmix (Novosibirsk, Russia)	95 °C for 3 min, (95 °C for 15 s, and 60 °C for 60 s) ×40.	
	5′-TTCCATTCTCCCCATCACTTTCA-3′								
	5′-FAM-GAGAAGGGAAGTTTAGAGAAAAAAGAAT-FQ-3′								
RNF219-For RNF219-Rev	5′-(Y1-12)GTGATTGTGGGTATAGTTATAAAA-3′	600	177		161	17	Hotstart PCR buffer with additional MgCl_2_ (final concentration: 5 mM), 1 mM dNTPs, and 0.65 units of Hotstart Taq polymerase	95 °C for 15 min (95 °C for 60 s, 58 °C for 45 s, and 72 °C for 60 s) ×50	
	5′-(X1-8)ACTACCCCCATCTCCCAAAA-3′								
KIAA1539-For KIAA1539-Rev	5′-(X1-8)AGGAAGGAGGAGATAAAGTGAT-3′	600	105		89	5			
	5′-(Y1-12)CCCCTCTAAACTTATCATCACA-3′								
GSTP1-For GSTP1-Rev	5′-(Y1-12)ATTTGGGAAAGAGGGAAAGGTT-3′	600	158		142	17			
	5′-(X1-8)CTCTTCTAAAAAATCC-3′								

**Table 3:** Barcodes information.

Barcodes	Forward or Reverse Primer the Exact Barcode Used for Each Target		
	RNF219	KIAA1539	GSTP1
X1: TAGATCGC, X2: CTCTCTAT, X3: TATCCTCT, X4: AGAGTAGA, X5: ACTGCATA, X6: AAGGAGTA, X7: CTAAGCCT, X8: CCTCTCTG	Reverse	Forward	Reverse
Y1: TCGCCTTA, Y2: CTAGTACG, Y3: TTCTGCCT, Y4: GCTCAGGA, Y5: AGGAGTCC, Y6: CATGCCTA, Y7: GTAGAGAG, Y8: CCTCTCTG, Y9: AGCGTAGC, Y10: CAGCCTCG, Y11: TGCCTCTT, Y12: TCCTCTAC	Forward	Reverse	Forward

**Table 4:** Comparison of PCa patients with a joint group including HD and BPH patients.

Gene, Position, and Status (C or T after Conversion)	*p*-Value × 3 × 1167	Means (%)	Sensitivity for a 100% Specificity (%)	Specificity for a 100% Sensitivity (%)	Cutoff (%)	CV Accuracy (%)	CV Sensitivity (%)	CV Specificity (%)	CV AUC (% (DeLong’s CI)
PSA	0.016		10.0	55.6		75.0	65.0	80.6	83.9 (73.5, 94.2)
GSTP1.C9 + RNF219.C2 + GSTP1.C2			100	100		100	100	100	100
GSTP1.C9 + RNF219.C2 + GSTP1.C16			100	100		100	100	100	100
GSTP1.C9 + RNF219.C2.T10			95.0	97.1		94.5	90.0	97.1	92.4 (83.2, 100)
GSTP1.C3.C9	0.00000073	8.03/0.0394	80.0	54.3	0.101	87.3	80.0	91.4	93.0 (85.1, 100)
GSTP1.C9	0.00000090	8.35/0.160	80.0	48.6	0.215	90.9	80.0	97.1	92.6 (83.2, 100)
GSTP1.C9.T17	0.0000011	4.04/0.143	80.0	37.1	0.198	89.1	80.0	94.3	93.1 (83.5, 100)
GSTP1.T2.C9	0.0000011	3.71/0.142	75.0	45.7	0.177	87.3	80.0	91.4	93.0 (84.7, 100)
GSTP1.T1.C9	0.0000024	3.94/0.142	75.0	34.3	0.199	89.1	80.0	94.3	92.1 (82.2, 100)
GSTP1.T6.C9	0.0000024	3.71/0.142	70.0	51.4	0.177	87.3	80.0	91.4	92.6 (84.4, 100)
GSTP1.T4.C9	0.0000029	3.71/0.142	75.0	51.4	0.179	87.3	80.0	91.4	92.1 (83.1, 100)
GSTP1.C9.T16	0.0000050	4.01/0.140	70.0	48.6	0.190	89.1	85.0	91.4	92.0 (83.4, 100)
GSTP1.C9.T14	0.0000070	3.90/0.138	70.0	34.3	0.190	87.3	85.0	88.6	91.9 (82.3, 100)
GSTP1.T5.C9	0.0000084	3.67/0.143	75.0	42.9	0.188	87.3	80.0	91.4	91.7 (82.9, 100)
GSTP1.C9.C13	0.0044	4.76/0.0197	70.0	40.0	0.0512	85.5	70.0	94.3	85.0 (73.0, 97.0)

The mean values are reported as the mean in the PCa group/mean in the HD + BPH group. Sensitivity is the proportion of PCa patients correctly identified as such, specificity is the proportion of HD and BPH patients correctly identified as such. Abbreviations: CV = cross validation, AUC = ROC area under curve, CI = confidence interval.

**Table 5:** Comparison of BPH patients with the joint group including HD and PCa patients.

Option Number	Gene, Position, and Status (C or T after Conversion)	*p*-Calue × 3 × 1167	Means (%)	Sensitivity for a 100% Specificity (%)	Specificity for a 100% Sensitivity (%)	Cutoff (%; Ratio)	CV Accuracy (%)	CV Sensitivity (%)	CV Specificity (%)	CV AUC (%; DeLong’s CI)
	PSA	>1		0	12.8		55.4	23.5	69.2	54.9 (38.8, 71.0)
1	RNF219.C1.C2	0.000000000071	0.00500/0.133	100	100	0.0526 (1.03)	100	100	100	100
2	RNF219.C2.C4	0.000000000071	0.0101/0.206	100	100	0.0567 (1.20)	100	100	100	100
3	RNF219.C1.C5	0.000000000071	0.00986/0.136	100	100	0.0526 (1.03)	100	100	100	100
4	RNF219.C2.C5	0.000000000071	0.00528/0.160	100	100	0.0481 (1.24)	100	100	100	100
5	RNF219.C4.C5	0.000000000071	0.0199/0.144	100	100	0.0561 (1.31)	100	100	100	100
76	RNF219.C12.C17	0.000000000071	0.00464/0.135	100	100	0.0508 (1.38)	100	100	100	100
77	RNF219.C13.C17	0.000000000071	0.00650/0.139	100	100	0.0557 (1.15)	100	100	100	100
78	GSTP1.T7.C16	0.00000000010	4.10/0.26	100	100	1.32 (1.20)	100	100	100	100
79	GSTP1.T11.C16	0.00000000010	5.33/0.231	100	100	1.24 (1.70)	100	100	100	100
80	GSTP1.C4.C5	0.00000000010	0.0197/3.72	100	100	0.0548 (1.03)	100	100	100	100

The mean values are reported as the mean in the BPH group/mean in the HD + PCa group. Sensitivity is the proportion of BPH patients correctly identified as such, specificity is the proportion of HD and PCa patients correctly identified as such. Abbreviations: CV = cross validation, AUC = ROC area under curve, CI = confidence interval.

**Table 6:** Comparison of HDs with a joint group including patients with prostate tumors (BPH + PCa).

Gene, Position, and Status (C or T after Conversion)	*p*-Value × 3 × 1167	Means (%)	Sensitivity for a 100% Specificity (%)	Specificity for a 100% Sensitivity (%)	Cutoff (%; Ratio)	CV Accuracy (%)	CV Sensitivity (%)	CV Specificity (%)	CV AUC (%; DeLong’s CI)
PSA	0.0000000076		26.3	97.3		98.2	100	97.3	97.3 (92.0, 100)
GSTP1.T3.T13	0.000000000049	99.5/88.5	100	100	99.3 (1.00)	100	100	100	100
GSTP1.T8.T13	0.00000000019	99.6/91.7	88.9	97.3	99.4	96.4	94.4	97.3	94.1 (86.1, 100)
GSTP1.T9.T13	0.0000000068	99.6/90.5	77.8	91.9	99.3	92.7	94.4	91.9	97.6 (94.3, 100)
GSTP1.C13	0.000000018	0.260/7.48	77.8	89.2	0.506	90.9	94.4	89.2	96.8 (93.2, 100)
GSTP1.T6.T13	0.000000018	99.5/91.9	77.8	89.2	99.2	90.9	94.4	89.2	97.7 (94.8, 100)
GSTP1.T1.C13	0.000000025	0.238/5.09	77.8	89.2	0.497	90.9	94.4	89.2	96.4 (92.4, 100)
GSTP1.T1.C6	0.000000078	0.188/.780	77.8	64.9	0.242	89.1	88.9	89.2	95.5 (89.8, 100)
GSTP1.C13.T17	0.000000078	0.241/5.15	77.8	83.8	0.496	87.3	94.4	83.8	96.7 (92.7, 100)

The mean values are reported as the mean in the HD group/mean in the BPH + PCa group. Sensitivity is the proportion of HDs correctly identified as such, specificity is the proportion of BPH and PCa patients correctly identified as such. Abbreviations: CV = cross validation, AUC = ROC area under curve, CI = confidence interval.

## Data Availability

The data presented in this study are available in this article (and supplementary material).

## Supplementary Materials

The following are available online at https://www.mdpi.com/article/10.3390/cancers13246234/s1; Table S1: Total data of a comparison of PCa patients with a joint group including HD and BPH patients. Sensitivity is the proportion of PCa patients correctly identified as such, specificity is the proportion of HD and BPH patients correctly identified as such; Table S2: Total data of a comparison of BPH patients with a joint group including HD and PCa patients. Sensitivity is the proportion of BPH patients correctly identified as such, specificity is the proportion of HD and PCa patients correctly identified as such; Table S3: Total data of a comparison of healthy donors with a joint group including patients with prostate tumors (BPH + PCa). Sensitivity is the proportion of HDs correctly identified as such, specificity is the proportion of BPH and PCa patients correctly identified as such; Table S4: Comparison of methylation patterns in the normal and the cancer groups WGBS samples; Table S5: Methylation patterns variables established in comparison of healthy donors with a joint group including patients with prostate tumors (targeted BS-seq) and their occurrence in the normal and the cancer groups WGBS samples comparison; Table S6: Methylation patterns variables established in comparison of PCa patients with a joint group including BPH and healthy donors (targeted BS-seq) and their occurrence in the normal and the cancer groups WGBS samples comparison.
