# Supplementary material for: Locus-Specific Methylation of GSTP1, RNF219, and KIAA1539 Genes with Single Molecule Resolution in Cell-Free DNA from Healthy Donors and Prostate Tumor Patients: Application in Diagnostics
Source: Cancers (Basel). 2021 Dec 12;13(24):6234. doi: 10.3390/cancers13246234 (PMC8699300; doi:10.3390/cancers13246234)
Supplement: Supplementary file 1 [file cancers-13-06234-s001.zip › Suppl_Table S4_WGBS.pdf]

Supplementary Table S4. Comparison of methylation patterns in the normal and the cancer group WGBS samples.

	P-value x561	Positive in normal group	Positive in cancer group
**GSTP1.T16.T17**	.00000000000000024[a]	54.0% (27 of 50)	4.6% (10 of 219)
**GSTP1.T14.T15**	.0000000000000014[a]	50.0% (24 of 48)	3.6% (8 of 220)
**GSTP1.T14.T16**	.000000000000016[a]	47.8% (22 of 46)	2.6% (5 of 193)
**GSTP1.T12.T13**	.000000000000017[a]	49.1% (27 of 55)	4.8% (11 of 229)
**GSTP1.C16**	.000000000000065[a]	46.2% (24 of 52)	93.6% (205 of 219)
**GSTP1.T15.T16**	.000000000000093[a]	48.9% (23 of 47)	3.6% (7 of 196)
**GSTP1.T11.T13**	.00000000000016[a]	49.0% (25 of 51)	4.9% (11 of 223)
**GSTP1.T14.T17**	.00000000000022[a]	47.7% (21 of 44)	3.1% (6 of 193)
**GSTP1.T11.T12**	.00000000000048[a]	49.0% (25 of 51)	5.6% (13 of 234)
**GSTP1.T12.T14**	.0000000000036[a]	45.5% (20 of 44)	1.4% (2 of 148)
**GSTP1.T13.T15**	.000000000047[a]	46.7% (21 of 45)	2.7% (4 of 149)
**GSTP1.T9.T13**	.00000000018[a]	52.3% (23 of 44)	6.6% (12 of 182)
**GSTP1.T13.T14**	.00000000029[a]	46.7% (21 of 45)	3.8% (6 of 158)
**GSTP1.C11**	.00000000053[a]	52.8% (28 of 53)	92.3% (227 of 246)
**GSTP1.T9.T11**	.00000000086[a]	50.0% (23 of 46)	7.3% (15 of 205)
**GSTP1.T10.T13**	.0000000010[a]	49.0% (24 of 49)	6.8% (13 of 190)
**GSTP1.T7.T13**	.0000000012[a]	56.1% (23 of 41)	7.3% (11 of 151)
**GSTP1.T10.T11**	.0000000013[a]	47.1% (24 of 51)	7.0% (15 of 213)
**GSTP1.C12**	.0000000017[a]	50.9% (28 of 55)	90.8% (218 of 240)
**GSTP1.T13.T16**	.0000000023[a]	44.2% (19 of 43)	1.6% (2 of 122)
**GSTP1.T11.T14**	.0000000029[b]	45.0% (18 of 40)	1.4% (2 of 142)
**GSTP1.T9.T12**	.0000000035[a]	52.3% (23 of 44)	8.3% (16 of 193)
**GSTP1.T8.T13**	.0000000052[a]	54.8% (23 of 42)	7.8% (12 of 154)
**GSTP1.T10.T12**	.0000000056[a]	49.0% (24 of 49)	8.0% (16 of 201)
**GSTP1.T11.T15**	.0000000075[b]	45.0% (18 of 40)	1.5% (2 of 133)
**GSTP1.C14**	.000000020[a]	50.0% (24 of 48)	90.4% (207 of 229)
**GSTP1.T12.T15**	.000000029[a]	45.5% (20 of 44)	4.3% (6 of 139)
**GSTP1.T7.T11**	.000000032[a]	53.5% (23 of 43)	9.2% (16 of 174)
**GSTP1.T7.T12**	.000000048[a]	56.1% (23 of 41)	9.9% (16 of 162)
**GSTP1.T12.T16**	.000000050[a]	42.9% (18 of 42)	1.8% (2 of 112)
**GSTP1.C11.C12**	.000000066[a]	51.0% (26 of 51)	89.7% (210 of 234)
**GSTP1.T6.T11**	.000000079[a]	64.3% (18 of 28)	10.3% (15 of 146)
**GSTP1.T6.T13**	.000000088[b]	69.2% (18 of 26)	8.1% (10 of 123)
**GSTP1.T6.T12**	.00000020[a]	69.2% (18 of 26)	11.9% (16 of 134)
**GSTP1.T8.T11**	.00000029[a]	52.3% (23 of 44)	10.2% (18 of 177)
**GSTP1.T9.T14**	.00000037[b]	48.5% (16 of 33)	2.0% (2 of 101)
**GSTP1.T8.T12**	.00000044[a]	54.8% (23 of 42)	10.9% (18 of 165)
**GSTP1.T13.T17**	.00000070[a]	43.9% (18 of 41)	4.1% (5 of 122)
**GSTP1.C14.C16**	.00000074[a]	52.2% (24 of 46)	90.7% (175 of 193)
**GSTP1.T11.T16**	.0000019[b]	42.1% (16 of 38)	1.9% (2 of 106)
**GSTP1.C11.C14**	.0000032[a]	55.0% (22 of 40)	93.7% (133 of 142)
**GSTP1.T6.T14**	.0000042[b]	73.3% (11 of 15)	0% (0 of 42)
**GSTP1.T11.T17**	.0000044[b]	41.7% (15 of 36)	1.9% (2 of 106)
**GSTP1.T8.T14**	.0000052[a]	51.6% (16 of 31)	2.7% (2 of 73)
**GSTP1.T7.T14**	.0000054[a]	53.3% (16 of 30)	2.9% (2 of 70)

**GSTP1.T10.T14**	.0000063[a]	44.7% (17 of 38)	4.6% (5 of 109)
**GSTP1.T15.T17**	.0000074[a]	48.9% (22 of 45)	11.2% (22 of 196)
**GSTP1.C11.C16**	.000013[a]	57.9% (22 of 38)	96.2% (102 of 106)
**GSTP1.C13**	.000018[a]	50.0% (28 of 56)	84.9% (203 of 239)
**GSTP1.T7.T8**	.000024[a]	50.0% (28 of 56)	14.8% (32 of 216)
**GSTP1.C13.C14**	.000041[a]	53.3% (24 of 45)	89.9% (142 of 158)
**GSTP1.T5.T13**	.000044[b]	66.7% (14 of 21)	9.1% (9 of 99)
**GSTP1.T12.T17**	.000044[a]	42.5% (17 of 40)	5.4% (6 of 112)
**GSTP1.T9.T10**	.000054[a]	47.3% (26 of 55)	13.9% (32 of 230)
**GSTP1.T7.T10**	.000061[a]	50.0% (26 of 52)	14.6% (29 of 199)
**GSTP1.C13.C16**	.000067[a]	55.8% (24 of 43)	92.6% (113 of 122)
**GSTP1.C12.C16**	.000090[a]	57.1% (24 of 42)	93.8% (105 of 112)
**GSTP1.T7.T9**	.000094[a]	50.9% (27 of 53)	15.8% (32 of 203)
**GSTP1.T10.T15**	.00011[a]	44.7% (17 of 38)	6.0% (6 of 100)
**GSTP1.C17**	.00013[a]	44.2% (23 of 52)	80.4% (181 of 225)
**GSTP1.C12.C14**	.00019[a]	54.5% (24 of 44)	89.9% (133 of 148)
**GSTP1.T5.T12**	.00019[b]	66.7% (14 of 21)	11.3% (12 of 106)
**GSTP1.T8.T9**	.00020[a]	50.0% (27 of 54)	16.0% (33 of 206)
**GSTP1.T6.T8**	.00032[a]	56.1% (23 of 41)	17.6% (33 of 188)
**GSTP1.T10.T16**	.00034[a]	41.7% (15 of 36)	2.7% (2 of 73)
**GSTP1.T8.T10**	.00042[a]	49.1% (26 of 53)	15.8% (32 of 202)
**GSTP1.C8**	.00045[a]	50.9% (29 of 57)	83.1% (182 of 219)
**GSTP1.T6.T10**	.00047[a]	56.8% (21 of 37)	17.0% (29 of 171)
**GSTP1.T6.T9**	.00059[a]	57.9% (22 of 38)	18.3% (32 of 175)
**GSTP1.T9.T16**	.00059[a]	45.2% (14 of 31)	3.1% (2 of 65)
**GSTP1.T3.T4**	.00073[a]	50.0% (21 of 42)	15.2% (32 of 210)
**GSTP1.T4.T5**	.00076[a]	55.0% (22 of 40)	17.6% (34 of 193)
**GSTP1.C11.C13**	.00086[a]	51.0% (26 of 51)	83.4% (186 of 223)
**GSTP1.C5**	.00098[a]	44.2% (19 of 43)	80.4% (176 of 219)
**GSTP1.C6.C12**	.0012[a]	30.8% (8 of 26)	79.1% (106 of 134)
**GSTP1.C16.C17**	.0013[a]	46.0% (23 of 50)	79.9% (175 of 219)
**GSTP1.T5.T8**	.0013[a]	52.8% (19 of 36)	15.0% (24 of 160)
**GSTP1.T9.T15**	.0013[a]	48.5% (16 of 33)	8.7% (8 of 92)
**GSTP1.T5.T9**[*]	.0014[a]	54.5% (18 of 33)	15.0% (22 of 147)
**GSTP1.T5.T11**	.0017[b]	60.9% (14 of 23)	12.7% (15 of 118)
**GSTP1.C15**	.0019[a]	49.0% (24 of 49)	81.6% (182 of 223)
**GSTP1.T5.T6**	.0020[a]	52.5% (21 of 40)	17.2% (35 of 203)
**GSTP1.C7.C12**	.0025[a]	43.9% (18 of 41)	80.9% (131 of 162)
**GSTP1.C7.C8**	.0026[a]	50.0% (28 of 56)	81.0% (175 of 216)
**GSTP1.C8.C12**	.0031[a]	45.2% (19 of 42)	81.2% (134 of 165)
**GSTP1.T2.T3**	.0034[a]	48.8% (21 of 43)	16.3% (35 of 215)
**GSTP1.T4.T13**	.0038[b]	66.7% (12 of 18)	12.0% (9 of 75)
**GSTP1.T2.T4**	.0040[a]	48.8% (20 of 41)	15.5% (29 of 187)
**GSTP1.T6.T7**	.0044[a]	53.5% (23 of 43)	19.6% (42 of 214)
**GSTP1.C6.C8**	.0055[a]	43.9% (18 of 41)	79.3% (149 of 188)
**GSTP1.C12.C13**	.0055[a]	50.9% (28 of 55)	80.8% (185 of 229)
**GSTP1.C12.C15**	.0056[a]	54.5% (24 of 44)	87.1% (121 of 139)
**GSTP1.C6.C11**	.0058[a]	35.7% (10 of 28)	78.8% (115 of 146)
**GSTP1.C8.C11**	.0062[a]	47.7% (21 of 44)	81.4% (144 of 177)
**GSTP1.C2**	.0062[a]	48.8% (21 of 43)	81.4% (180 of 221)

**GSTP1.C7**	.0063[a]	50.0% (29 of 58)	79.3% (192 of 242)
**GSTP1.C10**	.0064[a]	53.3% (32 of 60)	81.5% (194 of 238)
**GSTP1.T5.T7**	.0064[a]	50.0% (19 of 38)	16.1% (30 of 186)
**GSTP1.T4.T12**	.0080[b]	66.7% (12 of 18)	13.8% (11 of 80)
**GSTP1.C7.C11**	.0081[a]	46.5% (20 of 43)	80.5% (140 of 174)
**GSTP1.T8.T15**	.0089[a]	51.6% (16 of 31)	9.4% (6 of 64)
**GSTP1.T5.T10**	.011[a]	53.1% (17 of 32)	16.1% (23 of 143)
**GSTP1.C10.C12**	.011[a]	49.0% (24 of 49)	80.1% (161 of 201)
**GSTP1.C5.C12**	.012[a]	33.3% (7 of 21)	81.1% (86 of 106)
**GSTP1.T5.T14**	.013[b]	70.0% (7 of 10)	0% (0 of 24)
**GSTP1.C11.C15**	.014[a]	55.0% (22 of 40)	87.2% (116 of 133)
**GSTP1.C6.C9**	.015[a]	42.1% (16 of 38)	77.7% (136 of 175)
**GSTP1.T4.T8**	.017[a]	51.5% (17 of 33)	15.7% (21 of 134)
**GSTP1.C9**[*]	.017[a]	51.8% (29 of 56)	79.9% (187 of 234)
**GSTP1.T3.T5**	.020[a]	52.6% (20 of 38)	19.3% (36 of 187)
**GSTP1.C6**	.022[a]	44.4% (20 of 45)	76.2% (176 of 231)
**GSTP1.T7.T15**	.027[a]	53.3% (16 of 30)	11.5% (7 of 61)
**GSTP1.C10.C11**	.029[a]	51.0% (26 of 51)	79.8% (170 of 213)
**GSTP1.T3.T13**	.030[b]	62.5% (10 of 16)	11.4% (8 of 70)
**GSTP1.T6.T15**	.030[b]	73.3% (11 of 15)	12.1% (4 of 33)
**GSTP1.T2.T5**	.033[a]	51.4% (19 of 37)	18.3% (30 of 164)
**GSTP1.C6.C7**	.034[a]	41.9% (18 of 43)	74.3% (159 of 214)
**GSTP1.C9.C12**	.036[a]	47.7% (21 of 44)	78.8% (152 of 193)
**GSTP1.C7.C9**	.039[a]	49.1% (26 of 53)	77.8% (158 of 203)
**GSTP1.T4.T7**	.043[a]	48.6% (17 of 35)	16.2% (26 of 160)
**GSTP1.C8.C9**	.045[a]	50.0% (27 of 54)	78.2% (161 of 206)

[a] Chi-square test [b] Exact Fisher test [*] The corresponding difference was found to be statistically significant in the comparison of PCa patients and the group of HD and BPH.
